# Investigation of Tensile Creep Behavior for High-Density Polyethylene (HDPE) via Experiments and Mathematical Model

**DOI:** 10.3390/ma14206188

**Published:** 2021-10-18

**Authors:** Qiang Mao, Buyun Su, Ruiqiang Ma, Zhiqiang Li

**Affiliations:** 1 Institute of Applied Mechanics, College of Mechanical and Vehicle Engineering, Taiyuan University of Technology, Taiyuan 030024, China; maoqiang0016@link.tyut.edu.cn (Q.M.); subuyun@tyut.edu.cn (B.S.); maruiqiang@tyut.edu.cn (R.M.); 2 Shanxi Key Laboratory of Material Strength & Structural Impact, Taiyuan University of Technology, Taiyuan 030024, China; 3 National Demonstration Center for Experimental Mechanics Education, Taiyuan University of Technology, Taiyuan 030024, China; 4 College of Aeronautics and Astronautics, Taiyuan University of Technology, Taiyuan 030600, China

**Keywords:** high-density polyethylene (HDPE), uniaxial tensile experiment, creep behavior, time hardening form model

## Abstract

Temperatures of −25 °C, +5 °C, and +35 °C were selected to study the creep behavior of high-density polyethylene (HDPE). The ultimate tensile strength of HDPE materials was obtained through uniaxial tensile experiments and the time–strain curves were obtained through creep experiments. When the loaded stress levels were lower than 60% of the ultimate strength, the specimens could maintain a longer time in the stable creep stage and were not prone to necking. In contrast, the specimens necked in a short time. Then, the time hardening form model was applied to simulate the time–strain curve and the parameter values were solved. The parameter values changed exponentially with the stresses, thereby expanding and transforming the time hardening model. The expanded model can easily and accurately predict creep behaviors of the initial and stable creep stages as well as the long-term deformations of HDPE materials. This study would provide a theoretical basis and reference value for engineering applications of HDPE.

## 1. Introduction

Due to its advantages of a lightweight, low cost, high yield, and easy processing, the polymers are widely used in industry and daily life, especially in high-strength film, blow molding, injection molding, pipes, cables, and sheets [1]. With the development of science and technology, recycling has also become a trend in the use of the materials [2]. HDPE is a semi-crystalline polymer with high crystallinity, a milky translucent waxy solid, and its crystallinity is usually between 64% and 80% [3]. Because of the degree of the branched-chain is very small, the molecules can be stacked tightly, and the density is between 0.941–0.965 g/cm^3^, thus it has higher rigidity and toughness, good mechanical properties, and wider service temperatures [4].

However, polymer materials must maintain the loading state for a long time in the applications of daily life. For thermoplastic materials [5], the interactions between their linear molecular chains are weak, so that the mechanical properties of HDPE are poor, which limits the applications of HDPE. For example, it is easy to produce warpage deformations in the molding process, it is unable to bear heavy pressure and tension, poor mechanical properties at high temperatures, obvious aging phenomenon, and so on.

As a primary method to characterize viscoelasticity, creep is the time-dependent deformations of materials under constant stresses [6]. When the load is removed, creep will produce irrecoverable deformation and eventually lead to structural failure. Therefore, the creep test is a required method to control the bearing capacity of products in engineering applications. It is vital to understand the evolution processes of creep to predict the load-bearing capacity of the polymer structures and the deformations after long-term use.

Up until now, experts and scholars have conducted many studies on the properties and creep behaviors of HDPE materials. The creep model with two thermal activation processes proposed by Ward [7] is attractive and instructive. Ward [8] et al. pointed out that the deformation of HDPE fibers mainly included two aspects: the linear viscoelastic deformation and the non-recoverable nonlinear plastic flow changes. KWW (the Kohlrausch–Williams–Watts) equation [9] is widely used to analyze the creep behavior of glassy amorphous polymers, especially the physical aging of glassy amorphous polymers. It is considered that creep compliance increases exponentially with time, but the applications have obvious time limitations. The time–temperature equivalent principle can describe the viscoelastic behavior of amorphous polymers such as creep and stress relaxations, but it cannot effectively represent the creep behavior of the semi-crystalline high polymers. The theory and viscoelastic–plastic constitutive equations proposed by Schapery [10] can be widely used to evaluate the characteristics of the tested materials and verify the theory, but the theory and algorithm are complicated. In addition, several empirical mathematical models are often used to formulate the creep behaviors of polymers. For example, in the four-element Burger model [11], by connecting a Maxwell unit and a Kelvin unit in series, the Burger model divides the creep strain of polymer material into three parts: (1) the instantaneous deformation (Maxwell spring); (2) viscoelastic deformation (Kelvin unit); and (3) the viscous deformation (Maxwell dash-pot) [12]. The Findley power–law model [13] is also a widely used model to describe the creep deformations of polymers and composites. The Monkman–Grant relationship is another useful creep model that has been widely applied to metallic materials [14]. Although these empirical models could simulate the creep curves of polymers, it takes many experiments to determine the long-term creep behaviors of polymers, which requires a lot of time.

In this study, the uniaxial tensile behavior with different stretching rates and the creep behavior with different stress levels under three temperatures (−25 °C, +5 °C, and +35 °C) were studied. Moreover, the time–strain curves of the creep experiments were simulated based on the time hardening model. The parameter values were solved in the model and the changing trends of the parameter values with stress levels were analyzed. According to the variation tendencies, the parameter values loaded to other stress levels can be calculated quickly, thereby expanding the model. The curves drawn by the expanded model in comparison with the test curves and the curves simulated by the time hardening model results were in good agreement.

## 2. Uniaxial Tensile Experiment

### 2.1. Experimental Program

**Figure 1:**
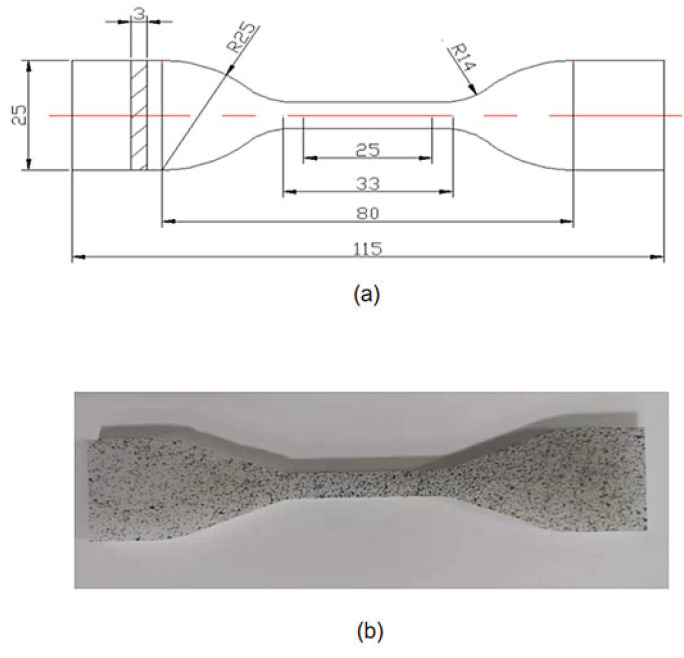
(**a**) Schematic diagram of the size of the specimen for uniaxial tensile experiment. (**b**) Physical image of specimen with speckle.

**Figure 2:**
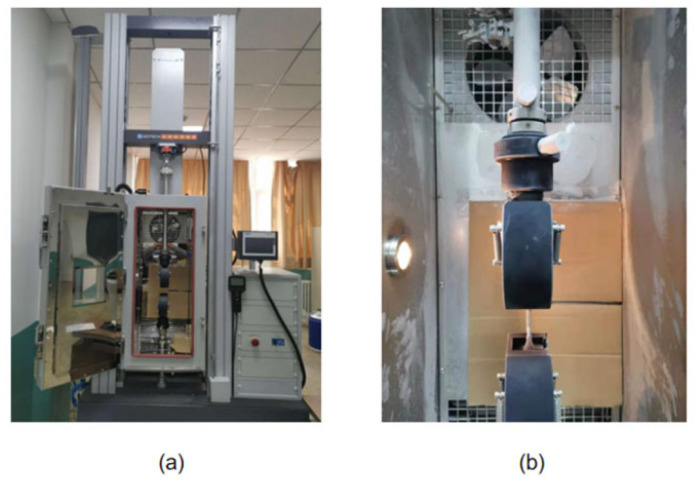
(**a**) Experimental machine. (**b**) Temperature control box.

HDPE specimens were used in this experimental study to investigate the ultimate strength at different temperatures. The dog-bone specimens were obtained from the extruded pipes and cut according to the specimen size. These pictures of the specimen and experimental machine are shown in Figure 1 and Figure 2, respectively. The schematic diagram of the specimen size is shown in Figure 1a and the physical image of the specimen sprayed with speckles is shown in Figure 1b. Figure 2a,b displays the tensile testing machine (GOTECH, Taiwan, China) and temperature control box (GOTECH, Taiwan, China) during the experiments.

Uniaxial tensile experiments were carried out on standard specimens at different ambient temperatures and the stretching rates at each temperature were 1.5 mm/s and 15 mm/s, respectively. During the experiments, the maximum loads were recorded and the deformations measured by DIC. By processing the experimental data, the stress–strain curves of HDPE materials were obtained at −25 °C, +5 °C, and +35 °C. The ultimate tensile strength of HDPE was obtained by the stress–strain curves.

### 2.2. Analysis of Experimental Data

**Figure 3:**
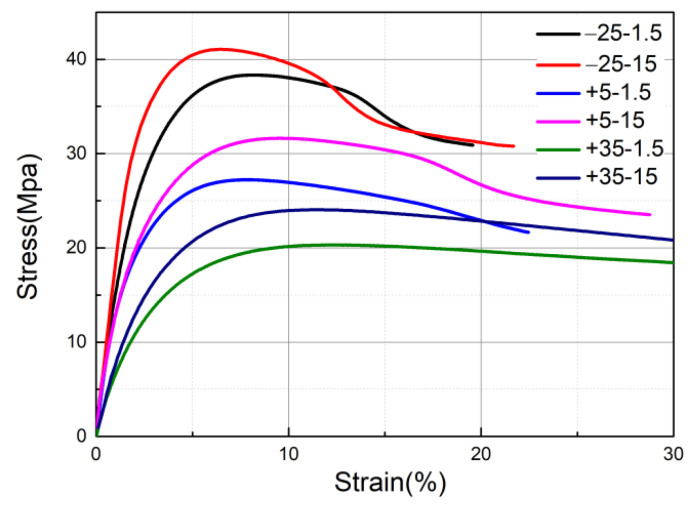
Stress–strain curves at different temperatures and tensile rates.

The curves of the load with displacement were obtained by the experimental machine (GOTECH, Taiwan, China). The speckles were sprayed on the specimen, and the strain variations with stress were calculated by DIC technology. The stress–strain curves of different tensile rates at three temperatures (−25 °C, 5 °C, and 35 °C) are shown in Figure 3. Strain rate and temperature have a great influence on the mechanical properties of HDPE materials [15].

- Influence of strain rate

In the stress–strain curves of Figure 3, by comparing curves with two different stretching rates, the following results can be obtained: First, at T = −25 °C, when the specimens were in the elastic phase, the stress–strain curves increased linearly. Under the same strain, the stresses increased more at the stretching rate of 15 mm/s than that at the stretching rate of 1.5 mm/s. When the curve reached the peak, the curve began to decline. The peak stress of the curve with a stretching rate of 15 mm/s was higher than that of the stretching rate of 1.5mm/s, and when the curve with a stretching rate of 15 mm/s reached the peak, its strain was lower than the stretching rate of 1.5 mm/s. Taking the peak value of the curve with a stretching rate of 1.5 mm/s as the ultimate tensile strength [15] at this temperature, the ultimate stretching strength of HDPE at T = −25 °C was 38.33871 MPa. The same can be obtained that at T = +5 °C it is 27.23671 MPa and at T = +35 °C, it is 20.31775 MPa. Second, from the stress–strain curves, it can be seen that the elastic modulus and yield stress of HDPE materials have a great relationship with the strain rate. The HDPE materials exhibited coupling viscoelastic deformation behavior after being stressed [16].

- Influence of temperature

Comparing the curves with stretching rates of 15 mm/s in Figure 3, at T = −25 °C, the strain when the stress reached the ultimate tensile strength was 5%; at T = +35 °C, the strain when the stress reached the ultimate tensile strength was 10%. At T = −25 °C, the curve had an obvious peak; at T = +35 °C, the curve tended to be flat after reaching the ultimate tensile strength. Moreover, the ultimate tensile strength at T = −25 °C was much higher than at T = +35 °C. Thus, HDPE materials are susceptible to temperature and the properties are greatly affected by the temperatures.

## 3. Creep Experiment

The rearrangement of molecules causes the creep deformations under long-time static loading, the initially curled molecular chains will stretch and slip, and the intermolecular force will be destroyed [17,18]. Unlike plastic deformation, creep failure generally occurs when the stress is far below the ultimate tensile strength, which can be at any temperature, even room temperature. The important influencing factors include material type, stress size, temperature, and time. In addition, to reduce the cost and test time, the long-term creep behavior analysis can be inferred from the short-term experimental data, accelerating the formation of creep failure at high temperatures or high tensile stress.

**Figure 4:**
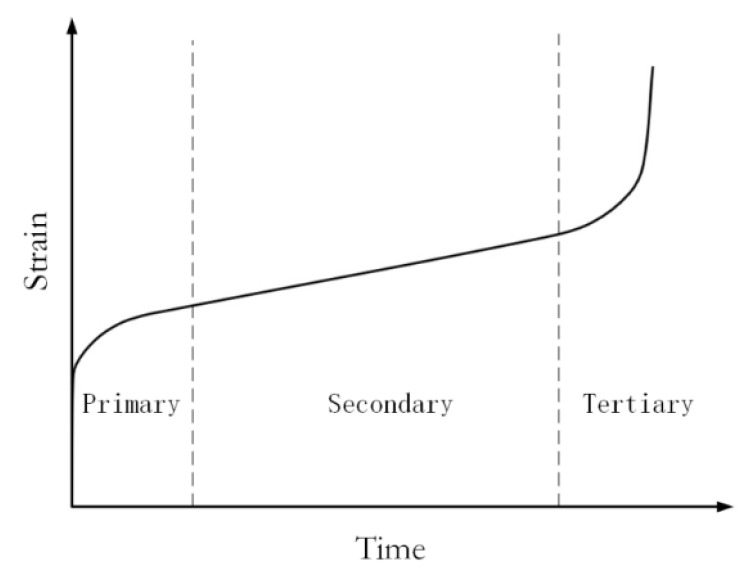
The three creep stages of the polymers.

Creep behavior is roughly divided into three stages [19,20]. The trend of the time–strain curve is shown in Figure 4. The initial stage is known as deceleration creep or instantaneous creep. In this phase, the molecules inside the crystal undergo an amorphous rearrangement, and the elongation is recoverable. The strain increases rapidly with time, but the strain rate decreases gradually. The second stage is called steady creep. Relative sliding between molecular chains and the elongation is irreparable. The strain increases linearly over time, while the strain rate keeps constant. The third stage is referred to as accelerated creep, in which the internal structure between the molecular chains has changed, resulting in the destruction of intermolecular forces. Hence, the strain increases sharply with time until the creep failure of the materials occur.

### 3.1. Experimental Program

**Figure 5:**
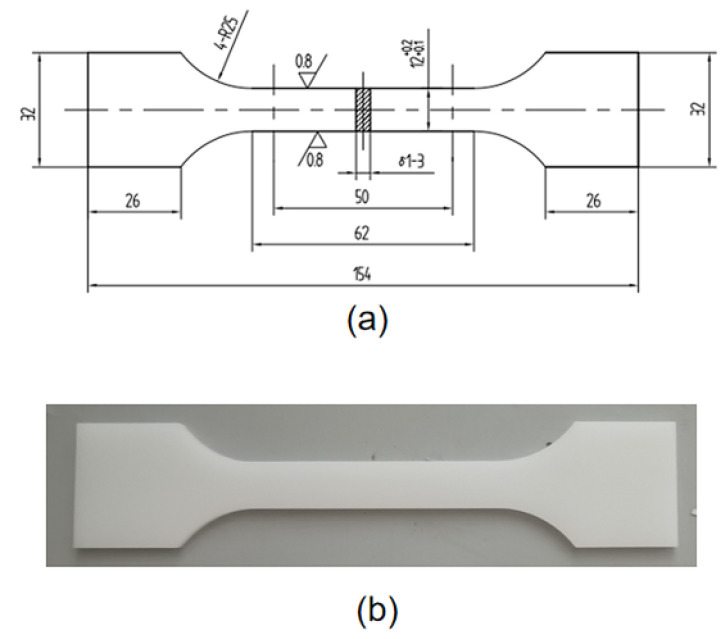
(**a**) Schematic diagram of the size of the specimen for the creep experiment. (**b**) Physical image of the specimen.

**Figure 6:**
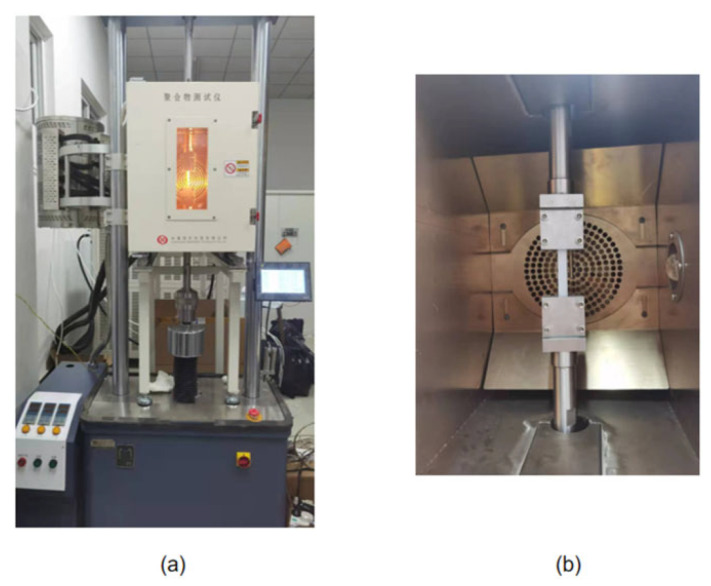
(**a**) Experimental machine. (**b**) Temperature control box.

A series of isothermal uniaxial tensile creep experiments are performed by loading HDPE specimens and simultaneously recording the changes of the strain over time. The dog-bone specimens were obtained from the extruded pipes and cut according to the size of the specimen, and the thickness of the test specimens was 3 mm. These pictures of the specimen and experimental machine are shown in Figure 5 and Figure 6, respectively. The schematic diagram of the specimen size is shown in Figure 5a and the physical image of the specimen is shown in Figure 5b. Figure 6a,b displays the creep machine (Kexin, Changchun, China) and temperature control box (Kexin, Changchun, China) during the experiments.

Three ambient temperatures (−25 °C, +5 °C, and +35 °C) were selected in the experiments. According to the ultimate strength of the specimens measured in the uniaxial tensile test, we selected the required five stress levels of the materials at each temperature. The specimens were subjected to constant axial stress under uniform temperature until necking occurred or kept for 12 h. The displacements of the specimens over time are recorded. Table 1 displays the test schemes [21,22].

For HDPE, the T_g_ = −110 °C, which is much lower than the experimental temperature. If the loaded stress is lower than the ultimate strength and the temperature is higher than T_g_, the creep behavior is theoretically controlled by the deformation in the amorphous phase. Therefore, the initial response stage of the material is the deformation of the amorphous phase [23,24].

### 3.2. Analysis of Experimental Data

**Figure 7:**
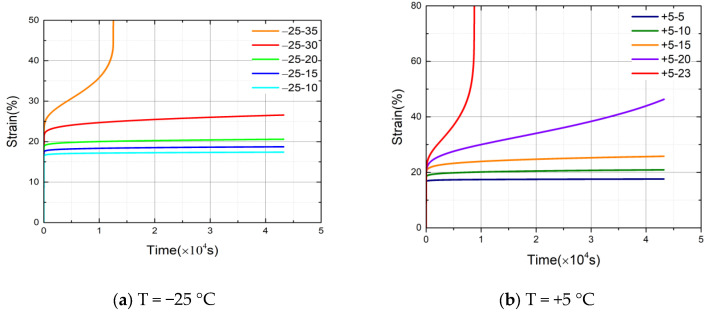

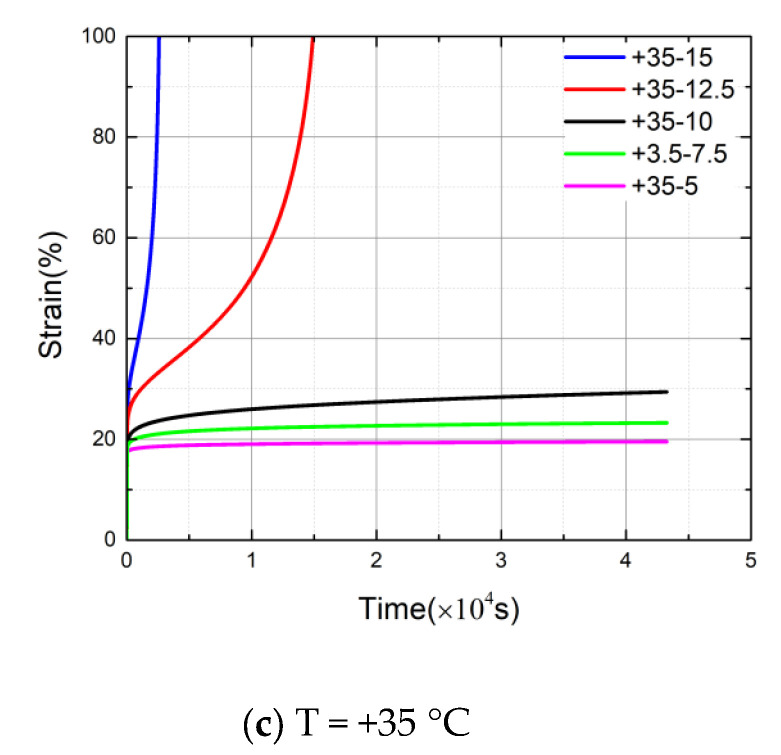
(**a**) Creep curves under different stress levels at T = −25 °C. (**b**) Creep curves under different stress levels at T = +5 °C. (**c**) Creep curves under different stress levels at T = +35 °C.

**Figure 8:**
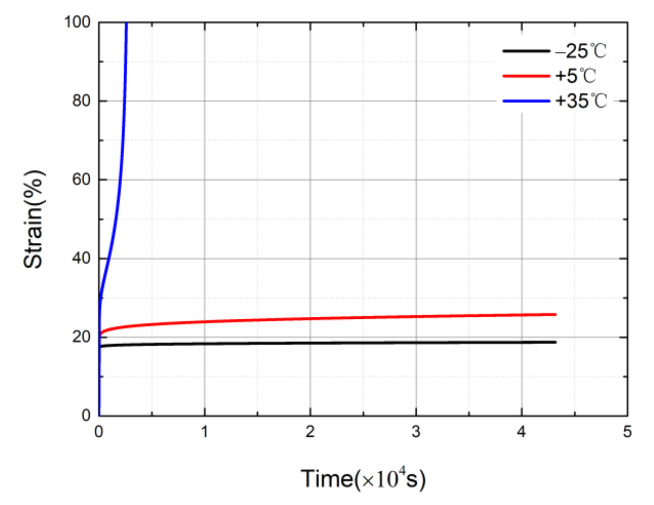
Creep curves at different temperatures under a loaded stress level of 15 MPa.

The time–strain curves are calculated and drawn based on the data of the displacement with time in the experiments, as shown in Figure 7 and Figure 8, respectively.

Figure 7 indicates the data curves under different loading stress levels at T = −25 °C, T = +5 °C, and T = +35 °C. First, the specimens necked after about 3 h when they were loaded with 35 MPa at T = −25 °C; the specimens necked after about 2 h when they were loaded with 23 MPa at T = +5 °C; and the specimens necked after about 0.7 h when they were loaded with 15 MPa at T = +35 °C. Thus, the specimens stayed in the stable creep stage for a very short time and entered the accelerated creep stage when they were loaded with stress closed to the ultimate strength of this temperature. The strain increased rapidly and the specimens shrank at that moment. Second, by comparing the curves that loaded different stress levels at the same temperature, the results indicate that the higher the stress levels of the specimens loaded, the longer the duration and the larger the strain in the initial stage. In contrast, the lower the stress levels of the specimens loaded, the faster the specimens entered the stable creep stage and the smaller the slopes of the curves in the stable creep stage, so the specimens remained longer in the stable creep phase. Third, from the three sets of time–strain curves at different temperatures, it can be found that when the stress levels of loading are below a certain value, the slopes of the curves in the stable creep stage is very small, so that the specimens remain longer in the stable creep stage. In contrast, the specimens will reach the accelerated creep state in a short time and neck. According to the curves, it can be preliminarily identified that the critical value [25] is 60% of the ultimate strength at a given temperature.

The data curves were acquired after being loaded with a stress of 15 MPa, as shown in Figure 8. The ambient temperatures were T = −25 °C, T = +5 °C, and T = +35 °C. It can be seen that temperature has a great effect on the creep behavior of HDPE. When T = +35 °C, the strain increased sharply in a short time and the specimens reached the accelerated creep stage. When T = −25 °C, the specimens quickly entered the stable creep stage and remained in the stable creep stage for a long time. Therefore, with the decrease in the ambient temperature, the earlier the specimens enter the stable creep stage, the lower the slopes of the time–strain curves, and the slower the strain growth in the stable creep stage. Moreover, the longer the residence time in the stable creep stage, the specimens are less likely to have necking occur.

## 4. Analysis of Creep Model

### 4.1. Creep Model

The mathematical models generally reflect the relationships between strain, stress, temperature, and rate, so selecting an accurate model for the finite element simulation is very important. At present, there are many mathematical models to describe the creep behavior of materials such as the power exponential function mathematical model and hyperbolic sine function mathematical model [26]. There are two types of power–law model creep models, one is the time hardening form, and the other is the strain hardening form [27].

For the strain hardening form, its mathematical model is as follows:

(1) $$\dot{\varepsilon }={\left\{A\sigma^{n}\left[\left(1+m\right)\varepsilon \right]\right\}}^{\frac{1}{1+m}},$$

where *σ* is the uniaxial equivalent stress; $\dot{\varepsilon }$ is the uniaxial equivalent creep strain; *A, m,* and *n* are parameters related to materials.

For the model of time hardening form, the ordinary creep process (when the loaded stress variation range is relatively small during the creep process) is more suitable, and its differential form is:

(2) $${\dot{\bar{\varepsilon }}}^{cr}=A{\tilde{q}}^{n}t^{m},$$

where $\dot{\bar{\varepsilon }}$ is the uniaxial equivalent creep strain rate; $\sqrt{\frac{2}{3}{\dot{\varepsilon }}^{cr}:{\dot{\varepsilon }}^{cr}}$, $\tilde{q}$ is the uniaxial equivalent deviatoric stress; *t* is the total or creep time; *A, m,* and *n* are constant terms, which are used to characterize the creep properties of the material.

Since the curves represent the relationship between creep strain and time and equivalent stress, Equation (2) must be integrated, and the results are as follows:

(3) $${\bar{\varepsilon }}^{cr}=\frac{A}{1+m}{\tilde{q}}^{n}t^{m+1},$$

where *n* > 0, 0 < *m* ≤ 1.

### 4.2. Mathematical Model Fitting

The curve fitting method is a common method to determine the creep parameters of HDPE. Based on the principle of the least square method, the relationship between strain and time obtained at different temperatures is introduced. The time hardening form model is selected to simulate and analyze the experimental curves, and then the material parameters in the model are solved. To make the fitting more convenient, the nonlinear creep equation is simplified, and the simplified equation is shown in Equation (4):

(4) $$\varepsilon \left(t\right)=a\times \sigma^{b}\times t^{c},$$

where *ε* is the uniaxial equivalent creep strain; *σ* is the uniaxial equivalent stress; *t* is the total or creep time; *a, b,* and *c* are constant terms, which are used to characterize the creep properties of the material.

**Figure 9:**
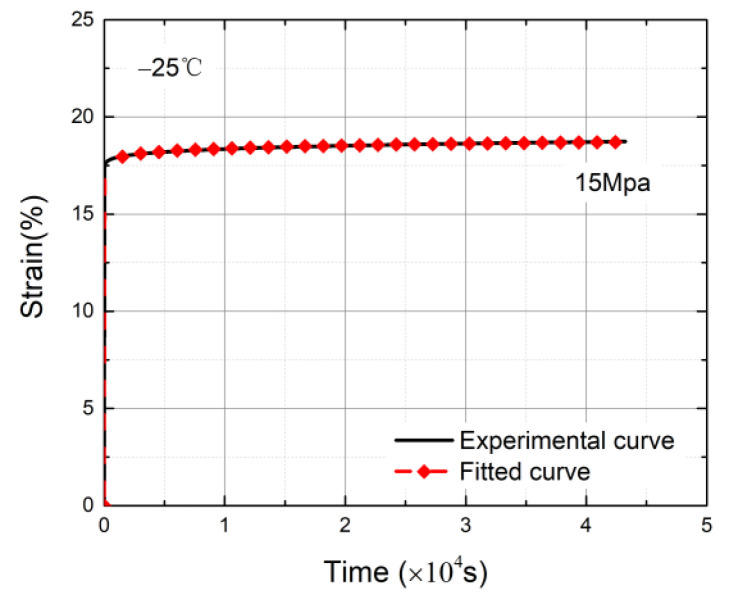
Curve fitting under a loaded stress level of 15 MPa at T = −25 °C.

The fitting curve is shown in Figure 9.

The parameters of the curve fitting are shown in Table 2.

From the comparison of the experimental data and fitting curve, the results indicate that the model could well describe the curve trend of creep in the initial and stable creep stages.

The model parameters fitted under other working conditions are shown in Table 3. Most of the Adj. R-Square representing the fitting results were above 0.95. Thus, Equation (4) well describes the curve trends in the initial and stable stages of creep.

### 4.3. Mathematical Model Analysis

**Figure 10:**
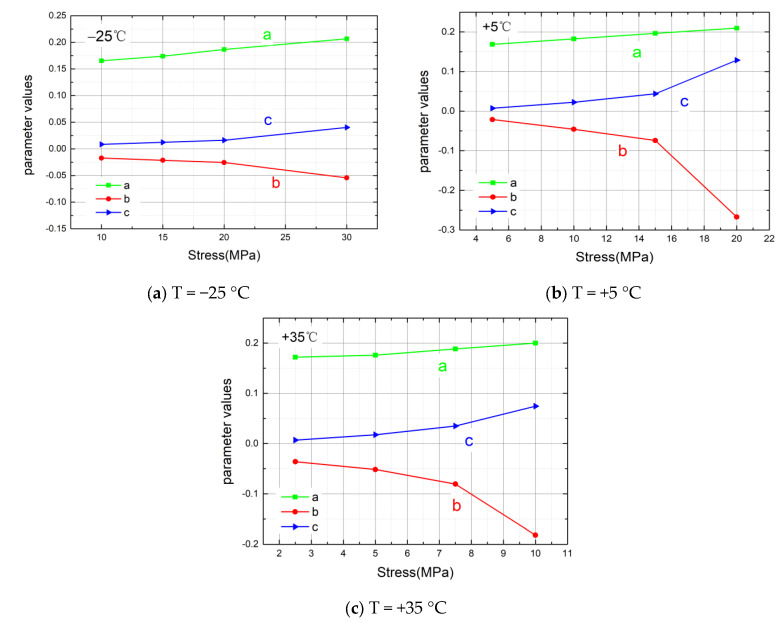
(**a**) Variation trend of model parameters with stress at T = −25 °C. (**b**) Variation trend of model parameters with stress at T = +5 °C. (**c**) Variation trend of model parameters with stress at T = +35 °C.

To emphasize the changes in the parameter values under different working conditions, the curves of the parameter values with stress levels are drawn, as shown in Figure 10.

According to the comparative analysis of the variation tendencies, as the loaded stress level increases, the values of parameter a and parameter c increase, but the values of parameter b decrease. Moreover, with the increase in the stress levels, the time–strain slopes of curves in the stable creep stage are higher, so the parameter values change more in Equation (4), and the changes in parameter b and parameter c are more obvious. The changing trends of the parameter values are similar to the exponential function.

**Figure 11:**
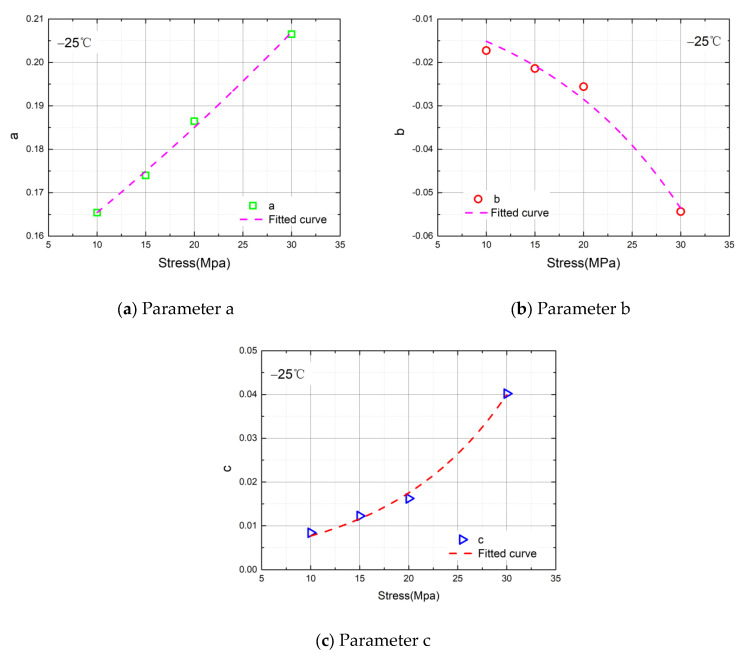
(**a**) Fitted curve of the value of parameter a when T = −25 °C. (**b**) Fitted curve of the value of parameter b when T = −25 °C. (**c**) Fitted curve of the value of parameter c when T = −25 °C.

The curves of the parameter values with the stress at T = −25 °C is shown in Figure 11.

When T = −25 °C, the fitting equations of the parameters are shown in Equation (5):

(5) $$\begin{array}{l} \mathrm{parameter}\;a:\;y=(0.1479\pm 0.00145)\times {\left(1.01126\pm 4.64E-4\right)}^{x}\\ \mathrm{parameter}\;b:\;y=(-0.00805\pm 0.00130)\times {\left(1.06258\pm 0.00653\right)}^{x}\\ \mathrm{parameter}\;c:\;y=(0.00336\pm 4.145E-4)\times {\left(1.08602\pm 0.00485\right)}^{x} \end{array}$$

The same can be obtained: when T = +5 °C, the fitting equations of the parameters are shown in Equation (6):

(6) $$\begin{array}{l} \mathrm{parameter}\;a:\;y=(0.1573\pm 9.88E-4)\times {\left(1.0146\pm 4.39E-4\right)}^{x}\\ \mathrm{parameter}\;b:\;y=(-0.00333\pm 0.00215)\times {\left(1.24456\pm 0.04124\right)}^{x}\\ \mathrm{parameter}\;c:\;y=(0.22245\pm 7.02E-4)\times {\left(1.21868\pm 0.0181\right)}^{x} \end{array}$$

when T = +35 °C, the fitting equation of the parameters is shown in Equation (7):

(7) $$\begin{array}{l} \mathrm{parameter}\;a:\;y=(0.16092\pm 0.0031)\times {\left(1.0215\pm 0.00275\right)}^{x}\\ \mathrm{parameter}\;b:\;y=(-0.01311\pm 0.00435)\times {\left(1.29785\pm 0.04644\right)}^{x}\\ \mathrm{parameter}\;c:\;y=(0.00375\pm 2.63E-4)\times {\left(1.34831\pm 0.01007\right)}^{x} \end{array}$$

Thus, the curves of the parameter values with the stress can be depicted by Equation (8):

(8) $$y=B_{i}\times C_{i}{}^{\sigma },$$

where *y* is the value of the parameter; *σ* is the uniaxial equivalent stress; *B_i_* and *C_i_* are constants related to the properties of the material and the loaded stress, respectively.

According to Equation (8), the model of the time hardening form can be transformed as follows:

$$\left\{ \begin{array}{l} {\bar{\varepsilon }}^{\mathrm{cr}}=\frac{A}{1+m}{\tilde{q}}^{n}t^{m+1}\\ \frac{A}{m+1}=B_{1}\times C_{1}^{\sigma },n=B_{2}\times C_{2}^{\sigma },m+1=B_{3}\times C_{3}^{\sigma } \end{array}\right.,$$

From simultaneous equations, it can be obtained that:

(9) $${\bar{\varepsilon }}^{\mathrm{cr}}=\left(B_{1}\times C_{1}^{\sigma }\right)\times \sigma^{B_{2}\times C_{2}^{\sigma }}\times t^{B_{3}\times C_{3}^{\sigma }},$$

Transform Equation (9) to obtain:

(10) $$\begin{array}{l} \ln{\bar{\varepsilon }}^{\mathrm{cr}}=\ln\left(\left(B_{1}\times C_{1}^{\sigma }\right)\times \sigma^{B_{2}\times C_{2}^{\sigma }}\times t^{B_{3}\times C_{3}^{\sigma }}\right)\\ \ln{\bar{\varepsilon }}^{\mathrm{cr}}=\ln\left(B_{1}\times C_{1}^{\sigma }\right)+\left(B_{2}\times C_{2}^{\sigma }\right)\ln\sigma +(B_{3}\times C_{3}^{\sigma })\ln t \end{array}$$

where ${\bar{\varepsilon }}^{cr}$ is the uniaxial equivalent creep strain; *σ* is the uniaxial equivalent stress; *t* is the total or creep time; and *B_i_* and *C_i_* are constants related to the properties of the material and the loaded stress, respectively.

On the basis of the tendencies of the parameter values with the loaded stress, by only doing three or four the creep experiments and calculating their parameter values, the parameter values loaded on other stress levels could be obtained at the same temperature. The tendencies could make it more convenient to solve the parameter values of the time hardening form model and predict the creep behaviors of the initial and the stable creep stages.

### 4.4. Results and Discussion

To verify the trends of the parameter values with the loaded stress, the experimental curves, the fitted curves of the original time hardening form model, and the expanded model curves were compared and analyzed.

**Figure 12:**
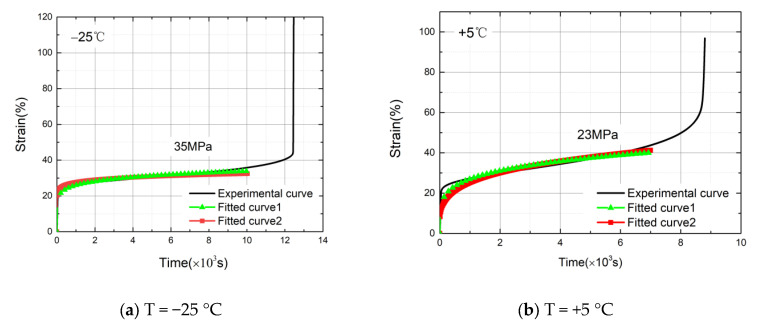

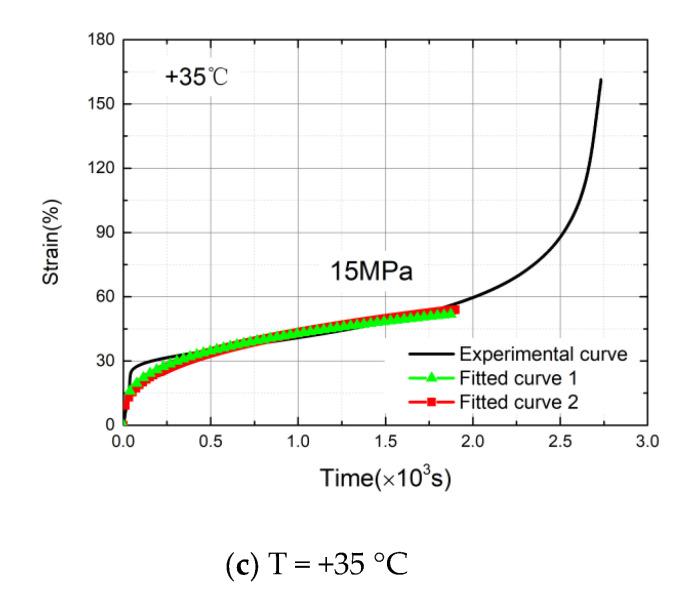
(**a**) Curve diagrams of 35 MPa stress at T = −25 °C. (**b**) Curve diagrams of 23 MPa stress at T = +5 °C. (**c**) Curve diagrams of 15 MPa stress at T = +35 °C.

According to Equation (5), the parameter values of 35 MPa stress at T = −25 °C were calculated. Similarly, from Equations (6) and (7), the parameter values of 23 MPa stress at T = +5 °C and 15 MPa stress at T = +35 °C were calculated. Under the same working conditions, the test curves, the fitted curves of the original time hardening model, and the expanded model curves were compared and analyzed. The curve diagrams are shown in Figure 12. The fitted curve 1 is the fitted curve by the original time hardening model to the experimental curve, and the fitted curve 2 is the curve drawn by the expanded model.

From the curve diagrams in Figure 12, the three curves coincided well. The curves drawn by the expanded model could accurately describe the fitted curves by the original time hardening model and the experimental curves. Thus, the expanded model could accurately and conveniently predict the initial and the stable creep stages as well as the long-term deformations of HDPE materials. For the accelerated creep stage, the strain hardening model may be considered.

## 5. Conclusions

This study is intended to investigate tensile creep behavior by creep experiments and expand the time hardening form model. The following conclusions can be made:

From the creep experimental curves, one can see that when the stresses of the specimens loaded were less than 60% of the ultimate tensile strength, the slopes of the time–strain curves were very small in the stable creep stage, thus the specimens remained for a longer time in the stable creep stage. Otherwise, the specimens necked in a short time.

The parameter values changed exponentially with the stress levels, thereby expanding and transforming the time hardening model. By conducting three to four creep experiments at a given temperature, the parameter values under other loaded stress levels can be calculated to predict the creep behaviors and deformations. According to the curve diagrams, the results reflect that the expanded model can depict the creep behaviors of the initial and stable creep stages as well as the long-term deformations of HDPE materials easily and accurately. This study has certain reference significance for the design of structural stability in engineering applications.

## Figures and Tables

**Table 1:** Experimental scheme.

Temperature (°C)	Ultimate Strength (MPa)	Stress Level (MPa)
−25	38.33871	35,30,20,15,10
+5	27.23671	23,20,15,10,5
+35	20.31775	15,12.5,10,7.5,5

**Table 2:** Curve fitting parameters.

Parameter	Value
a	0.17395
σ	10
b	−0.0214
c	0.01224

**Table 3:** Creep model parameters.

Temperature (°C)	Stress Level (MPa)	a	b	c	Adj. R-Square
−25	30	0.20651	−0.05437	0.04019	0.97488
	20	0.18647	−0.02558	0.01623	0.96932
	15	0.17395	−0.0214	0.01224	0.95829
	10	0.16541	−0.01726	0.00839	0.93505
+5	20	0.20957	−0.26719	0.12861	0.9453
	15	0.19628	−0.07403	0.04379	0.98563
	10	0.18232	−0.04578	0.02245	0.98041
	5	0.16847	−0.02116	0.00719	0.92029
+35	10	0.20013	−0.18177	0.07443	0.99125
	7.5	0.18848	−0.08045	0.03504	0.9963
	5	0.17595	−0.05154	0.01758	0.98574
	2.5	0.17193	−0.03621	0.00701	0.91014

## Data Availability

Not applicable.
